# The Impact of Ethnicity and Genetic Ancestry on Disease Prevalence and Risk in Colombia

**DOI:** 10.3389/fgene.2021.690366

**Published:** 2021-09-17

**Authors:** Aroon T. Chande, Shashwat Deepali Nagar, Lavanya Rishishwar, Leonardo Mariño-Ramírez, Miguel A. Medina-Rivas, Augusto E. Valderrama-Aguirre, I. King Jordan, Juan Esteban Gallo

**Affiliations:** ^1^ School of Biological Sciences, Georgia Institute of Technology, Atlanta, GA, United States; ^2^ IHRC-Georgia Tech Applied Bioinformatics Laboratory, Atlanta, GA, United States; ^3^ PanAmerican Bioinformatics Institute, Cali, Colombia; ^4^ National Institute on Minority Health and Health Disparities, National Institutes of Health, Bethesda, MD, United States; ^5^ Centro de Investigación en Biodiversidad y Hábitat, Universidad Tecnológica del Chocó, Quibdó, Colombia; ^6^ Biomedical Research Institute (COL0082529), Cali, Colombia; ^7^ Department of Biomedical Sciences, Universidad Santiago de Cali, Cali, Colombia; ^8^ Department of Biological Sciences, Universidad de los Andes, Bogotá, Colombia; ^9^ GenomaCES, Universidad CES, Medellín, Colombia

**Keywords:** Colombia, Latin America, health disparities, precision medicine, ethnicity, genetic ancestry, cancer, malaria

## Abstract

Currently, the vast majority of genomic research cohorts are made up of participants with European ancestry. Genomic medicine will only reach its full potential when genomic studies become more broadly representative of global populations. We are working to support the establishment of genomic medicine in developing countries in Latin America *via* studies of ethnically and ancestrally diverse Colombian populations. The goal of this study was to analyze the effect of ethnicity and genetic ancestry on observed disease prevalence and predicted disease risk in Colombia. Population distributions of Colombia’s three major ethnic groups – Mestizo, Afro-Colombian, and Indigenous – were compared to disease prevalence and socioeconomic indicators. Indigenous and Mestizo ethnicity show the highest correlations with disease prevalence, whereas the effect of Afro-Colombian ethnicity is substantially lower. Mestizo ethnicity is mostly negatively correlated with six high-impact health conditions and positively correlated with seven of eight common cancers; Indigenous ethnicity shows the opposite effect. Malaria prevalence in particular is strongly correlated with ethnicity. Disease prevalence co-varies across geographic regions, consistent with the regional distribution of ethnic groups. Ethnicity is also correlated with regional variation in human development, partially explaining the observed differences in disease prevalence. Patterns of genetic ancestry and admixture for a cohort of 624 individuals from Medellín were compared to disease risk inferred *via* polygenic risk scores (PRS). African genetic ancestry is most strongly correlated with predicted disease risk, whereas European and Native American ancestry show weaker effects. African ancestry is mostly positively correlated with disease risk, and European ancestry is mostly negatively correlated. The relationships between ethnicity and disease prevalence do not show an overall correspondence with the relationships between ancestry and disease risk. We discuss possible reasons for the divergent health effects of ethnicity and ancestry as well as the implication of our results for the development of precision medicine in Colombia.

## Introduction

Genomic medicine is an emerging medical discipline that entails the use of genomic information about an individual as part of their clinical care – in support of better diagnostic, prognostic, and therapeutic decision-making (Collins and Varmus, 2015; Jameson and Longo, 2015). Genomic medicine promises to revolutionize healthcare, but the vast majority of genomics research cohorts are currently made up of individuals with European ancestry (Bustamante et al., 2011; Popejoy and Fullerton, 2016). Clinical insights based on the study of European ancestry genomes will not necessarily replicate across diverse populations (Martin et al., 2017). This genomics research gap limits the reach of genomic medicine and threatens to exacerbate existing health disparities (Petrovski and Goldstein, 2016; Martin et al., 2019). The promise of genomic medicine will not be fully realized until genomic studies become more broadly representative of global populations.

Colombia has a diverse, multi-ethnic population with major ancestry contributions from Europe, Africa, and the Americas (Bryc et al., 2010; Ruiz-Linares et al., 2014; Homburger et al., 2015). Colombian genomic diversity represents a rich and largely untapped resource that can be used to support the development of genomic medicine locally in Colombia and around the world. We have been working to build local capacity in precision medicine *via* population and clinical genomic studies of diverse Colombian populations over the last decade (Rishishwar et al., 2015a,b; Jordan, 2016; Medina-Rivas et al., 2016; Chande et al., 2017, 2020a,b; Conley et al., 2017; Norris et al., 2018, 2020; Nagar et al., 2019). These studies share the broad aims of (1) characterizing patterns of genetic ancestry and admixture within and between Colombian and other Latin American populations, and (2) exploring the relationship between ancestry and genetic determinants of health and disease in the region.

The goal of the current study was to analyze the effect of ethnicity and genetic ancestry on observed disease prevalence and predicted disease risk in Colombia. We focused on Colombia’s three largest ethnic groups – Mestizo, Afro-Colombian, and Indigenous – along with corresponding genetic ancestry contributions from Europe, Africa, and the Americas. We studied health conditions and diseases that have been prioritized by the Colombian government as having an outsized impact on public health and the economy – six high impact non-cancer conditions along with eight of the most common cancers. Ethnicity and disease prevalence were analyzed at the level of administrative departments (states) and geographic regions, and genetic ancestry and disease risk were inferred using whole genome genotype (WGG) data for a diverse cohort of 624 individuals. We found that disease prevalence and risk are associated with ethnicity, geography, socioeconomics, and genetic ancestry in Colombia, and we discuss the implications of our findings with respect to the development of precision medicine in the country.

## Materials and Methods

### Study Cohorts

A cohort of 624 individuals from Medellín, Colombia was recruited and genotyped by GenomaCES Biotechnologies of the Universidad CES (UniCES),^1^ and a cohort of 99 individuals from Chocó, Colombia was recruited and genotyped as part of the ChocoGen research project.^2^ All sample donors signed informed consent, and all participant recruiting, sampling, and genetic characterization was done following the Helsinki ethical principles for medical research involving human subjects. Human subject research in Colombia was conducted in accordance with article 11, resolution 8,430, 1993 of the Colombian Ministry of Health, which states that for every investigation in which a human being is the study subject, respect for their dignity and the protection for their rights should always be observed. The UniCES Medellín cohort was recruited and characterized with the approval of the Ethics and Research Committee of the Universidad CES. The ChocoGen project was conducted with the approval of the Ethics Committee of the Universidad Tecnológica del Chocó. Population genomic analysis was approved by the Institutional Review Board of the Georgia Institute of Technology.

### Ethnicity, Disease Prevalence, and Socioeconomic Data

Data on Colombian ethnic groups was taken from the 2005 census as reported by the National Administrative Department of Statistics (DANE; Rojas Morales et al., 2007). Ethnic identity is self-reported in the Colombian census, and individuals choose from one of six ethnic groups: (1) *Indígena*, (2) *Rom*, (3) *Raizal del Archipiélago de San Andrés y Providencia*, (4) *Palanquero de San Basilio*, (5) *Negro(a)*, *mulato(a)*, *afrocolombiano(a) o afrodescendiente*, and (6) *Ninguna de las anteriores* (Supplementary Figure 1). We focused on the three largest ethnic groups in Colombia: Mestizo, Afro-Colombian, and Indigenous. The ethnic group labels, we use for this study are English translations of the officially used Spanish group names in Colombia, except for the Spanish word Mestizo, which we adopt here as it is widely used in both English and Spanish. Following the convention of DANE, Indigenous ethnic identity corresponds to question #1 *Indígena*, Afro-Colombian ethnic identity corresponds to questions #3, #4, and #5, all of which correspond to specific Afro-Colombian communities or identities, and Mestizo ethnic identity corresponds to question #6 *Ninguna de las anteriores* (none of the above). The Mestizo group, which may include individuals who identify as white or Mestizo, is also referred to as *sin pertenencia étnia* (no ethnicity), reflecting the fact that majority population individuals are not considered to belong to any of Colombia’s officially recognized minority ethnic groups. The *Rom* census question refers the Roma community, which makes up less than 0.01% of the Colombian population and was therefore not considered here. Population numbers and percentages for each of the three major ethnic groups are reported for the entire country and for each of 32 administrative departments plus the capital district of Bogotá. Regional population numbers and percentages for the three groups were calculated based on the administrative departments that make up each of the five geographic regions (Bustamente et al., 2012).

The Colombian *Instituto Nacional de Salud* and the non-governmental organization *Cuenta de Alto Costo* were used to identify health conditions and diseases that have a maximum impact on public health and the economy. We chose six non-cancer conditions and eight of the most common cancers for analysis. Prevalence data for these conditions and diseases were taken from two databases: (1) *Cuenta de Alto Costo*,^3^ and (2) *Sistema Nacional de Vigilancia en Salud Pública – SIVIGILA*.^4^ Disease prevalence values are calculated and expressed as age- and sex-adjusted prevalence per 100,000 population. Disease prevalence data are reported for the entire country and for each of 32 administrative departments plus the capital district of Bogotá.

The Human Development Index (HDI) is a composite index combining three dimensions of human development: long and healthy life, knowledge, and standard of living.^5^^,^ ^6^ Each dimension is measured by specific indicators: life expectancy at birth, expected years of schooling and mean years of schooling, and gross national income *per capita*. Indicators are normalized, using fixed maximum and minimum values for each indicator, to yield I_Health_, I_Education_, and I_Income_ dimension index values that range from 0 to 1: dimension index (I)=(actual indicator value – minimum indicator value)/(maximum indicator value – minimum indicator value). The arithmetic mean of the two normalized education dimension index values is calculated for I_Education_. The HDI is calculated as the geometric mean of the three dimension index values: HDI=(I_Health_×I_Education_×I_Income_)^1/3^. HDI data for Colombian administrative departments was taken from the Global Data Lab,^7^ and values range from 0.69 to 0.90.

Correlations between ethnic group percentages, HDI, and disease prevalence values for administrative departments were done using Pearson’s correlation (*R*) using the cor.test function in base R v3.5.1 (R Core Team, 2020). Ethnic group percentages are the percentage of the population that each ethnic group makes up for each administrative department, values range from 0 to 98.27%. Disease prevalence values are taken for each administrative department, as described above, values range from 0 to 7,847 cases per 100,000 population.

### Genetic Ancestry and Disease Risk Prediction

Universidad CES Medellín cohort participants’ WGG were characterized using the Illumina Global Screening Array,^8^ and ChocoGen cohort participants’ WGG were characterized using the Illumina HumanOmniExpress-24 Array.^9^ Full details on the sampling, DNA extraction, genotyping, and quality control procedures for these data, all of which were characterized for previous studies, have been reported elsewhere (Pato et al., 2013; Medina-Rivas et al., 2016; Conley et al., 2017; Bigdeli et al., 2020).

The UniCES Medellín cohort WGG data were merged and harmonized with whole genome sequence (WGS) data from global reference populations representing three continental population group ancestries – European, African, and Native American (Supplementary Table 1) – characterized as part of the 1,000 Genomes Project (1KGP), using the program PLINK v1.9 and bespoke scripts (1000 Genomes Project Consortium et al., 2015; Chang et al., 2015). Variant data from UniCES WGG and 1KGP WGS were merged to include variants that were present in both datasets with a missingness and minor allele frequency filters of 5 and 1%, respectively. Variant strand flips and identifier inconsistencies were corrected as needed. The merged and harmonized dataset contained 425,732 genome-wide variants. This combined dataset was then merged with WGG from Chocó, Colombia characterized as part of the ChocoGen project (see footnote 2), with a missingness threshold of 5%. The three-way merged UniCES-1KGP-ChocoGen dataset contained 77,575 variants. The three-way merged dataset was pruned for linkage disequilibrium (LD) using the “--indep” command in PLINK 1.9 with a window size of 50kb, a step size of five variants, and a variant inflation factor (VIF) threshold of two to yield a final merged, harmonized, and LD pruned dataset of 63,852 variants, which was used for genetic ancestry characterization.

Principal component analysis (PCA) of the final variant dataset was performed using PLINK using the “--pca” option and the first two PCs for all samples were plotted using the ggplot2 package in R v3.5.1 (Wickham, 2016; R Core Team, 2020). The program ADMIXTURE v1.30 was used to characterize participants’ genome-wide ancestry fractions for the three continental ancestry groups – European, African, and Native American (Alexander et al., 2009). ADMIXTURE was run in unsupervised mode with default settings and *K*=3.

The NHGRI-EBI genome-wide association study (GWAS) Catalog was mined for trait-variant associations that correspond to the health conditions and diseases studied here.^10^ Trait-associated variant sets corresponding to four of six non-cancer health conditions and seven of the eight common cancers were used to infer genetic disease risk *via* polygenic risk scores (PRS; Supplementary Table 2). GWAS were filtered by trait of interest, type of study, number of individuals and distinct ancestries in the discovery and replication cohorts, and finally by number of associations reported. We aimed to identify at least one large, multi-ethnic study for each trait, under the assumption that PRS derived from multi-ethnic cohorts are less likely to be biased. Only traits with at least 20 variant associations were retained for PRS calculation. Variant identifiers (rsid), effect alleles, effect sizes, and *p*-values were collected from for each selected trait-study combination. Curated variant lists for each trait were subjected to LD clumping (“--clump-r2 0.2”) and used to calculate PRS for imputed genomic variant data for the UniCES Medellín cohort using PLINK (v1.90b6.16), as previously reported (Chande et al., 2018, 2020a). PRS for each condition or disease *i* were calculated as the sum of the effect alleles across all trait-associated SNPs as – $PRS_{i}=\sum_{j=1}^{n}EA_{j}/\sum_{j=1}^{n}A_{j}$ – where $E_{j}\in \left\{0,1,2\right\}$ corresponds to homozygous absent, heterozygous present, or homozygous present effect alleles at each variant, and $A_{j}\in \left\{0,1,2\right\}$ corresponds to the total number of alleles with base calls at each variant. WGG data were imputed using the 1KGP haplotype reference panel with the program IMPUTE2 version 2.3.2 (Howie et al., 2011, 2012). Imputed sites were retained for subsequent analysis if they had a 95% imputation rate across samples and an INFO score >0.4.

Correlations between individuals’ genetic ancestry fractions and PRS were done using Pearson’s correlation (*R*) using the cor.test function in base R v3.5.1 (R Core Team, 2020). Genetic ancestry fractions range from 0 to 0.925, and PRS values range from 0 to 1.

## Results

### Ethnicity and Genetic Ancestry

**Figure 1:**
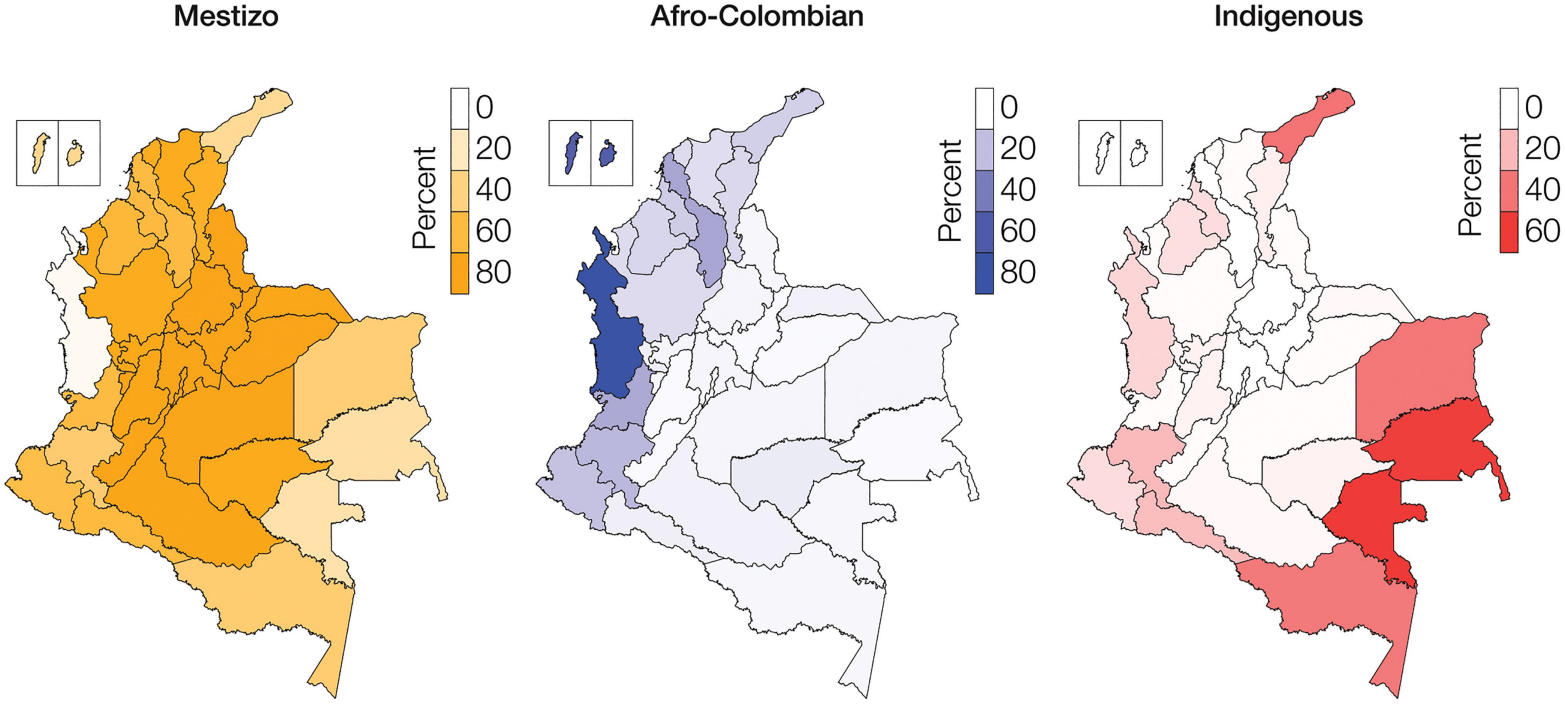
Ethnic groups geographic distributions. Percentages of Mestizo (orange), Afro-Colombian (blue), and Indigenous (red) populations among Colombia’s administrative departments.

The Colombian census reports the ethnic composition of the country as 85.9% Mestizo, 10.6% Afro-Colombian, and 3.4% Indigenous (Rojas Morales et al., 2007). There is high variation in the distribution of ethnic groups among Colombia’s administrative departments and geographic regions (Table 1; Supplementary Table 3). The percent Mestizo population of individual departments ranges from 5.2% in the department of Chocó in the western Pacífico region to 98.4% in the capital district of Bogotá. Afro-Colombian population percentages range from 1.0% for the department of Guainía in the eastern Amazonía region to 82.1% in Chocó. Indigenous percentages range from 0.1% on the Caribbean Islands of San Andrés and Providencia to 66.7% for the Vaupés department in the southeastern Amazonía region. Broadly speaking, Mestizo population composition is highest in the central Andes region, Afro-Colombian populations are concentrated in the Pacífico and Caribe regions along the Pacific and Atlantic coasts, and Indigenous populations are highest in the Amazonía region (Figure 1).

**Table 1:** Ethnic group population percentages for the five broad geographic regions and the entire country.

Region	Mestizo	Afro-Colombian	Indigenous
Amazonía	63.09	3.27	33.64
Andes	93.52	5.19	1.29
Caribe	70.33	19.13	10.53
Orinoquía	95.65	2.68	1.67
Pacífico	51.38	30.63	17.98
Total	85.94	10.62	3.43

**Figure 2:**
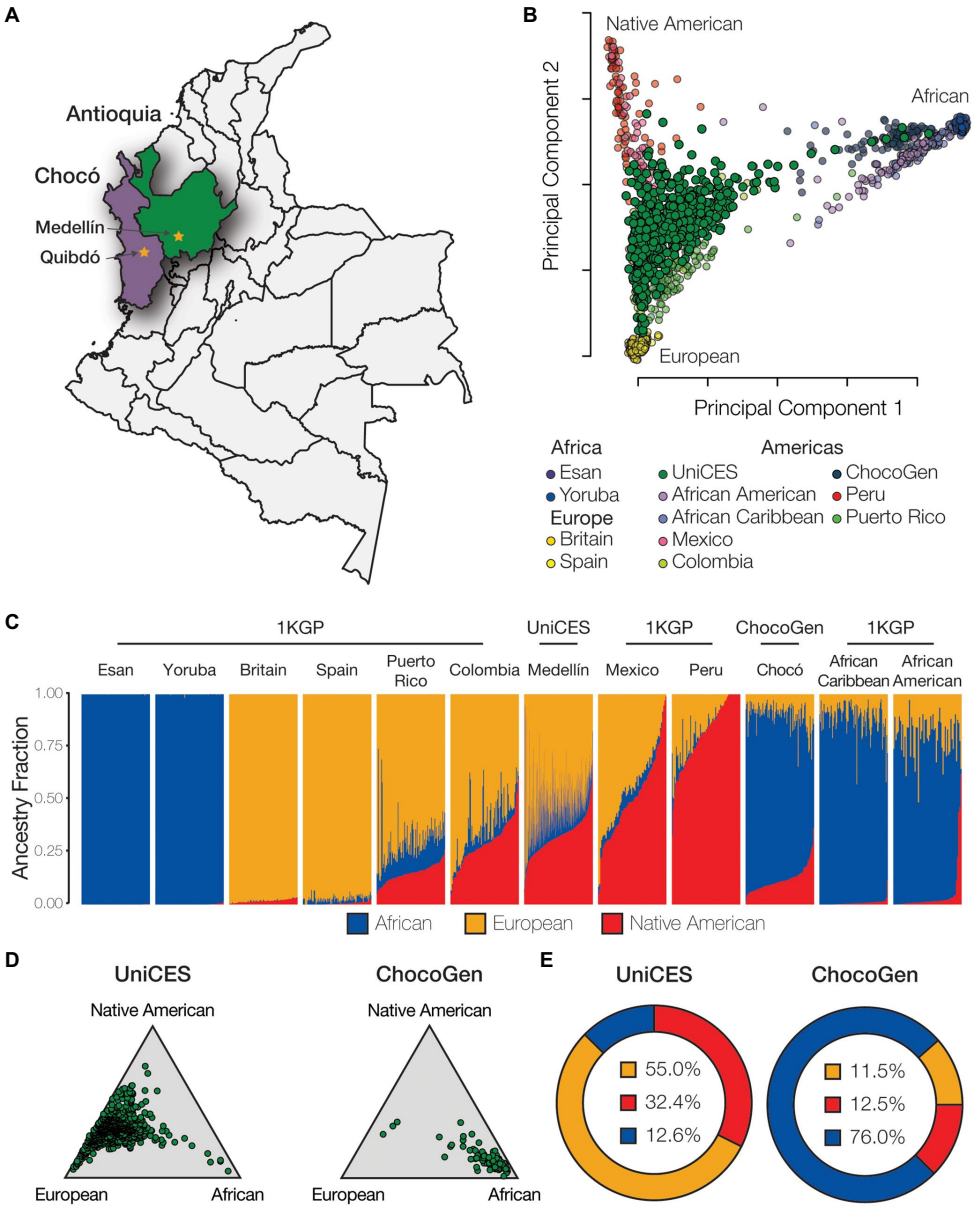
Genetic ancestry and admixture. **(A)** Cohort sampling locations in Medellín, Antioquia and Quibdó, Chocó. **(B)** Principal component analysis (PCA) showing the genetic relationships among individuals from admixed American populations, including the Colombian populations studied here, together with European, African, and Native American reference populations. **(C)** ADMIXTURE plot showing the patterns of genetic ancestry fractions – European (orange), African (blue), Native American (red) – for individuals from admixed American and global reference populations. **(D)** Ternary plots showing the relative distributions of continental ancestry for the Medellín and Chocó cohorts studied here. **(E)** Population average ancestry fractions for the for the Medellín and Chocó cohorts. Population abbreviations: 1KGP – 1,000 Genomes Project, ChocoGen – Chocoano in Quibdó, Colombia, UniCES – Cosmopolitan Colombian in Medellín, Colombia.

Whole genome genotype data were characterized for a cohort of 624 individuals sampled from the city of Medellín in the department of Antioquia and 99 individuals sampled from Quibdó in the department of Chocó (Figure 2A). Comparison of individual WGG data from these samples with WGS data from European, African, and Native American ancestry reference populations shows evidence of substantial admixture for all three continental ancestry components. The Medellín sample characterized here is similar, but substantially more ancestrally diverse, compared to the previously characterized 1KGP population from Colombia, and falls between reference populations corresponding to European, African, and Native American ancestry (Figure 2B). The Chocó sample is closer to the African American and African Caribbean 1KGP populations.

The program ADMIXTURE was used to quantify ancestry components for the Colombian genomes analyzed here. ADMIXTURE was run for *K*=2–10 ancestry components, with *K*=3 ancestry components showing the best fit to the data. ADMIXTURE analysis confirms the ancestral diversity of the two Colombian cohorts: the Medellín cohort shows an average of 55.0% European ancestry, 32.4% Native American ancestry, and 12.6% African ancestry, and the Chocó cohort shows an average of 76.0% African ancestry, 12.5% Native American ancestry, and 11.5% European ancestry (Figures 2C–E). All of the Colombian genomes analyzed here show evidence of admixture with two or more ancestry components, with a minimum non-European ancestry component of 7.5%. European ancestry percentages for individual Colombian genomes studied here range from 2.5 to 92.5%, Native American ancestry from 1.3 to 73.6%, and African ancestry from 0.0 to 92.1%, underscoring the diversity of Colombian populations.

### Ethnicity, Disease Prevalence, and Comorbidities

Department disease prevalence values, expressed as the age- and sex-adjusted number of cases per 100,000 individuals, were recorded for six health conditions and eight cancers prioritized as high impact by Colombian governmental and non-governmental organizations (Supplementary Table 4). Malaria shows the highest prevalence for the six prioritized non-cancer health conditions, with a per-department average of 618.5 cases per 100,000 individuals, followed by type 2 diabetes with 367.7 cases per 100,000. Breast cancer and prostate cancer were the most common cancer types, with prevalence values of 131.6 and 64.1 per 100,000 individuals, respectively. The highest combined prevalence for the six prioritized health conditions is seen for the departments of Guainía and Amazonas in the Amazonía region followed by Chocó in the Pacífico region. Disease prevalence in these departments is dominated by Malaria, but the combined disease prevalence remains high when Malaria is not considered. The Islands of San Andrés and Providencia are the exception to this trend with a high prevalence for non-Malaria conditions and little to no risk of Malaria. The highest combined prevalence across all eight cancers is seen for the departments of Risaralda, Antioquia, and the capital district of Bogotá, all of which are located in the central Andes region.

**Figure 3:**
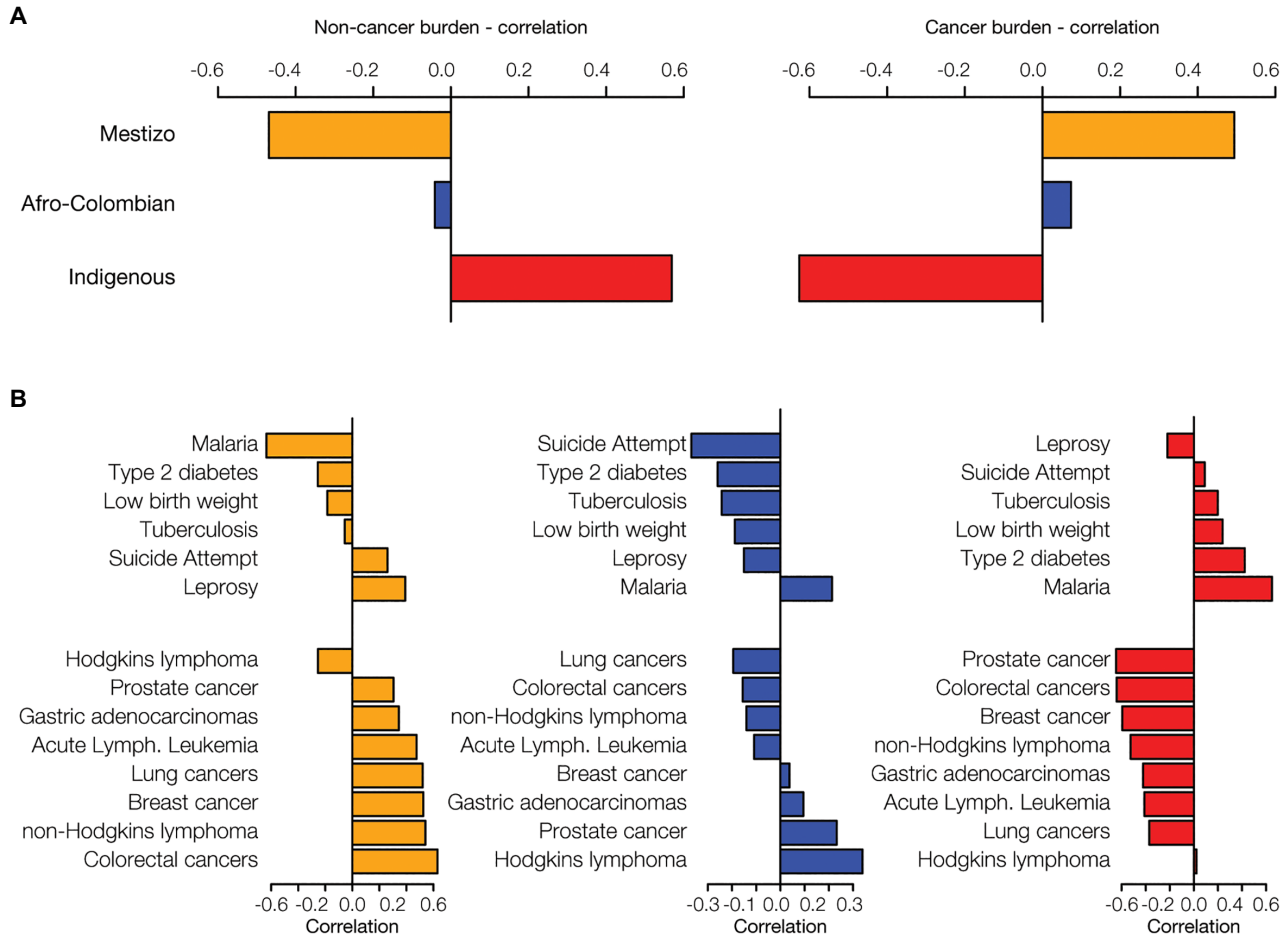
Ethnicity and disease prevalence. Administrative department ethnic group percentages were correlated with disease prevalence data. Data are shown for the three largest ethnic groups in Colombia: Mestizo (orange), Afro-Colombian (blue), and Indigenous (red). **(A)** The overall correlation of ethnicity with disease prevalence was calculated by summing the disease-specific correlations for six non-cancer health conditions and eight cancers. **(B)** For each ethnic group, ethnicity-disease prevalence correlations are shown for six non-cancer conditions (top of each plot) and the eight cancers (bottom of each plot).

Percentages of Mestizo, Afro-Colombian, and Indigenous ethnic populations across departments were compared to the disease prevalence data. The combined prevalence of the six prioritized non-cancer conditions is positively correlated with Indigenous population percentages and negatively correlated with Mestizo percentages (Figure 3A). Cancer shows the opposite pattern, a positive correlation with Mestizo population percentages and a negative correlation with Indigenous percentages. Afro-Colombian population percentages show comparatively smaller correlations with disease prevalence, only slightly negative for the non-cancer conditions and slightly positive for the cancers. Mestizo and Indigenous population patterns are mirror images of each other with respect to the prevalence of the individual non-cancer conditions and cancer (Figure 3B). Indigenous population percentages are positively correlated for six out of seven prioritized non-cancer conditions, whereas Mestizo and Afro-Colombian percentages are mostly negatively correlated with these same conditions. Malaria shows the highest overall correlations with ethnicity, with high positive correlations for both Afro-Colombian and Indigenous population percentages and the most extreme negative correlation with Mestizo percentages. Mestizo population percentages are positively correlated with seven out of eight cancer types and negatively correlated with Hodgkin’s Lymphoma, whereas Indigenous percentages show the opposite patterns.

**Figure 4:**
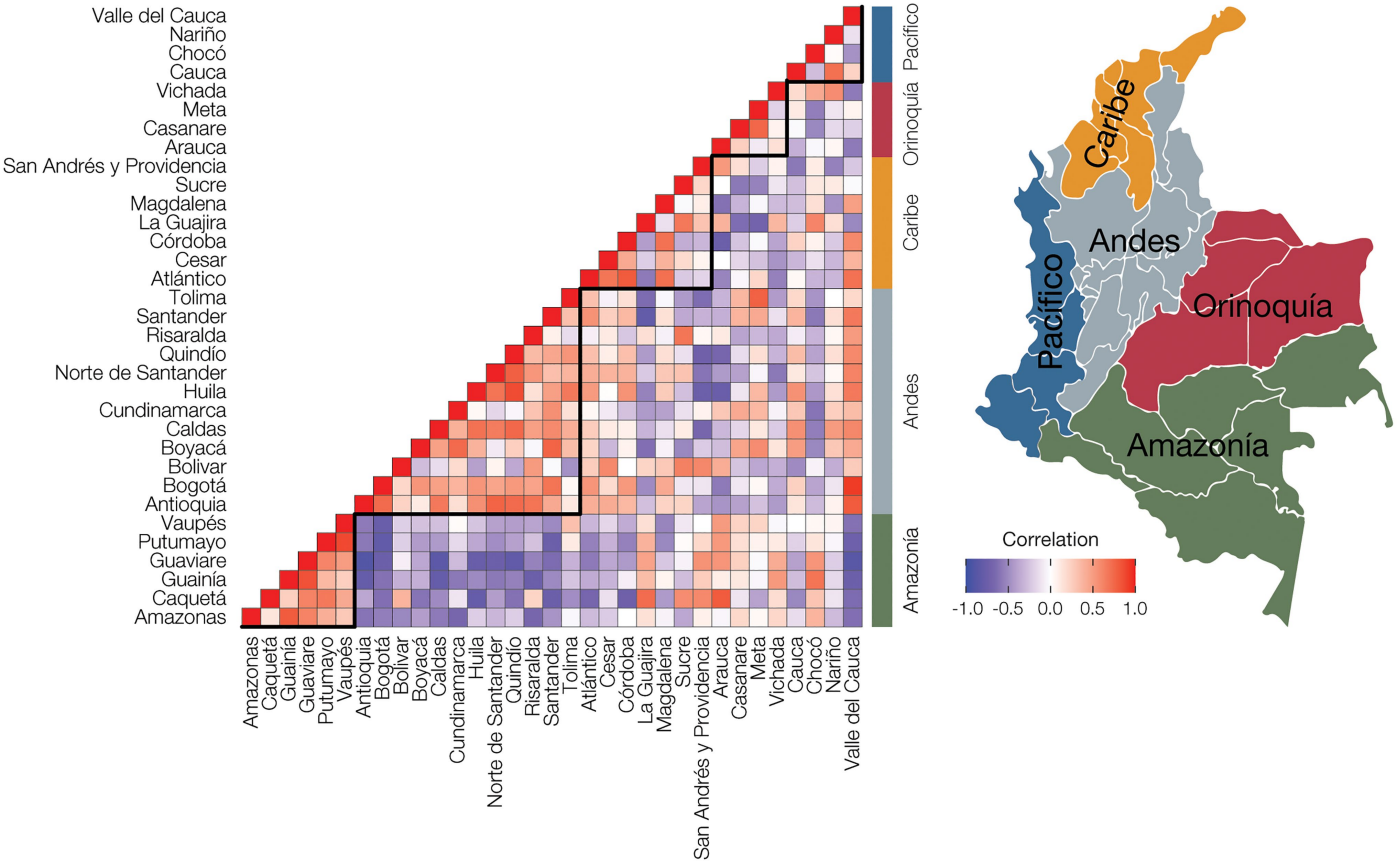
Population comorbidity and geography. Disease prevalence values were normalized for each administrative department by dividing the observed prevalence value for each disease with the maximum disease prevalence value for the department to yield relative disease prevalence values. The relative disease prevalence values were correlated between all pairs of departments to infer overall comorbidity levels, i.e., similar relative disease prevalence values, among departments and geographic regions. Positive disease prevalence correlations between departments are shown in red, and negative correlations are shown in purple. Administrative department names are shown to the left of the plot, and geographic region names are shown to the right of the plot, with locations indicated on the adjacent map.

Non-cancer and cancer disease prevalence values were normalized within departments (Supplementary Figure 2), and the resulting relative disease prevalence values for all departments were correlated to calculate pairwise disease comorbidities among all departments. The resulting departmental pairwise comorbidity values were hierarchically clustered and compared to the departments’ locations within Colombia’s five geographic regions (Figure 4). Population comorbidity values are highest around the diagonal line and correspond well with individual departments’ locations within broad geographic regions. The tightest clustering of comorbidity values, indicating similar relative disease prevalence values for departments within a given region, is seen for the Amazonía and Andes regions, corresponding to their high percentages of Indigenous and Mestizo populations, respectively. The Caribe region also shows high population comorbidity, whereas the comorbidity values for the Pacífico region are lower, reflecting greater demographic diversity for the region (Figure 1; Table 1; Supplementary Table 3).

### Genetic Ancestry and Predicted Disease Risk

Genetic risk estimates for four of the six of the prioritized non-cancer conditions and seven of the eight most common cancers were inferred using PRS computed on genomic variant data from the UniCES Medellín cohort studied here. Individuals’ genetic ancestry percentages – European, African, and Native American – were regressed against PRS for each trait to evaluate the relationship between ancestry and predicted disease risk.

**Figure 5:**
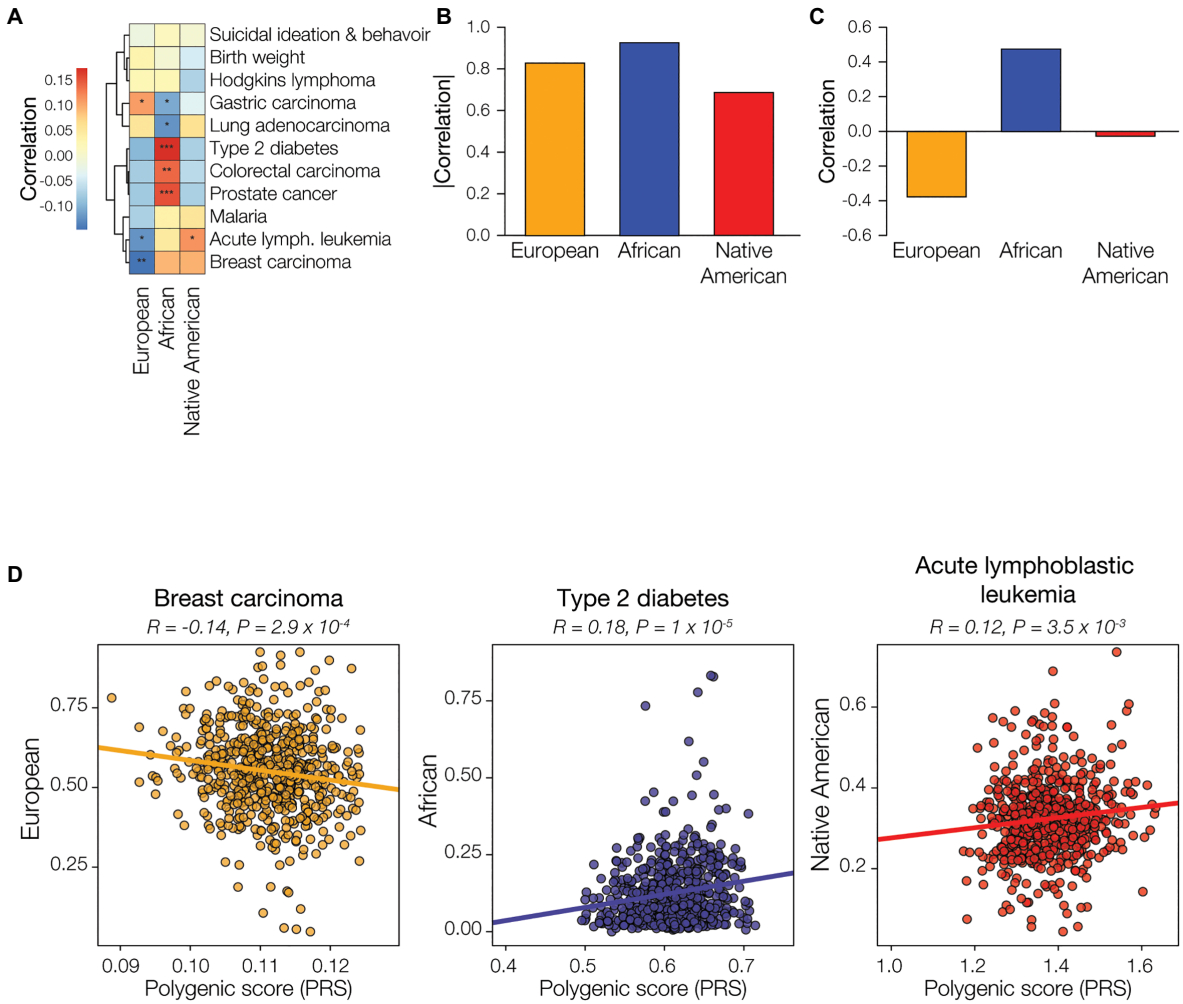
Genetic ancestry and disease risk. Genetic ancestry percentages – European (orange), African (blue), and Native American (red) – and disease polygenic risk scores (PRS) were computed from participant genomic data and correlated. **(A)** Examples of correlations between genetic ancestry and PRS are shown for each of the three ancestries. Pearson correlation coefficient and *p*-values are shown for each. **(B)** Genetic ancestry-PRS correlations for all three ancestries and 11 health conditions or diseases are shown. Positive correlations are shown in red, and negative correlations are shown in blue. Correlation statistical significance levels are indicated (0.01<*p*<0.075=*, 0.001<*p*<0.01=**, *p*<0.001=***). The overall correlation of genetic ancestry with disease risk was calculated by summing the disease-specific correlations, absolute values **(C)** and raw correlations **(D)**, for the 11 conditions.

European genetic ancestry is significantly positively correlated with the predicted risk of gastric carcinoma and negatively correlated with the risk of acute lymphoblastic leukemia and breast carcinoma (Figure 5A). African genetic ancestry shows statistically significant correlations with the predicted risk for five out of 11 diseases evaluated, compared to three and one significant correlation(s) for European ancestry and Native American, respectively. African genetic ancestry is positively correlated with the predicted risk of type 2 diabetes, colorectal carcinoma, and prostate cancer, and negatively correlated with gastric carcinoma and lung adenocarcinoma. The only significant correlation seen for Native American genetic ancestry is the negative correlation with the predicted risk for acute lymphoblastic leukemia. Overall, African genetic ancestry shows the highest effect on predicted disease risk, as measured by the sum of the absolute values of the ancestry-PRS correlations, followed by European ancestry and Native American ancestry, respectively (Figure 5B). African ancestry shows the highest overall positive correlation for predicted disease risk, as measured by the sum of the values of the ancestry-PRS correlations, whereas European ancestry shows the highest overall negative correlation with disease risk (Figure 5C). The overall correlation between Native American ancestry and predicted disease risk is negligible. Examples of regressions for individual ancestry-disease combinations are seen for European ancestry and breast carcinoma, African ancestry and type 2 diabetes, and Native American ancestry and acute lymphoblastic leukemia (Figure 5D).

### Differences Between Observed Disease Prevalence and Predicted Disease Risk

**Figure 6:**
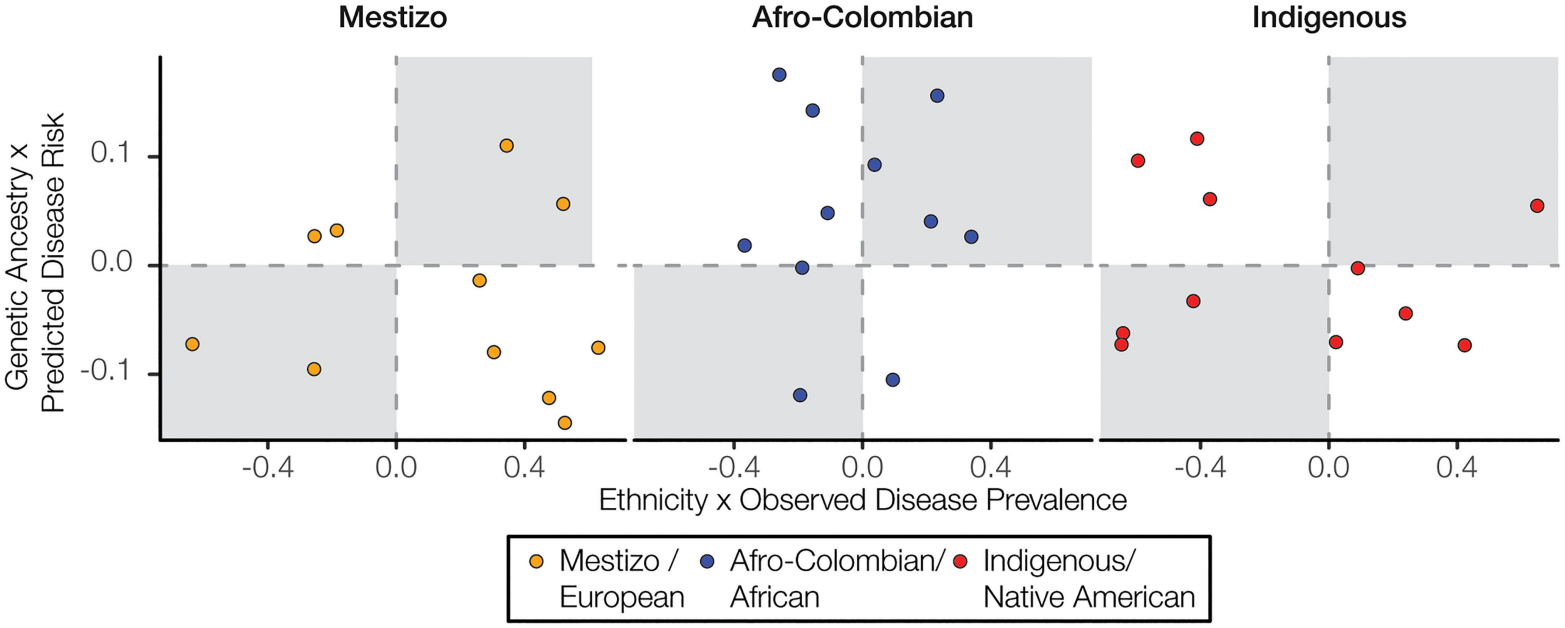
Ethnicity and disease prevalence vs. genetic ancestry and disease risk. Correlation values for ethnicity and disease prevalence are plotted against correlation values for genetic ancestry and disease risk. Concordant correlations, both positive or both negative, are shown in the upper right and lower left quadrants, respectively.

We analyzed the three major ethnic groups in Colombia – Mestizo, Afro-Colombian, and Indigenous – in comparison with three corresponding genetic ancestry groups – European, African, and Native American. Both ethnicity and genetic ancestry show a number of significant correlations with observed disease prevalence and predicted disease risk. However, the direction of the disease correlations for the corresponding ethnicity and ancestry groups often do not match. In other words, a positive (or negative) correlation between ethnicity and observed disease prevalence among departments does not necessarily entail a positive (or negative) correlation between genetic ancestry and predicted disease risk for the individuals in the ancestrally diverse cohort studied here, as may be expected given the correlation between ancestry and ethnicity in Colombia. For Afro-Colombian ethnicity and African genetic ancestry there are six concordant ethnicity/ancestry-disease correlation pairs and five discordant correlation pairs (Figure 6). For both Mestizo ethnicity-European genetic ancestry and Indigenous ethnicity-Native American genetic ancestry, there are four concordant correlation pairs and seven discordant pairs.

### Human Development, Ethnicity, and Health

**Figure 7:**
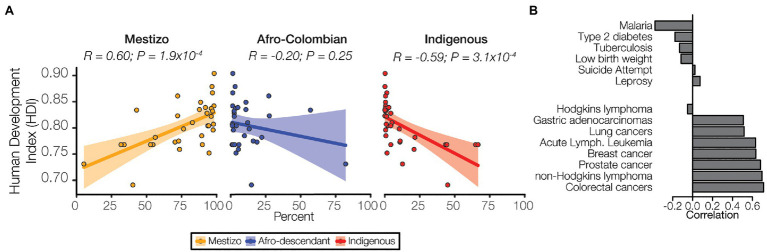
Ethnicity, socioeconomic status, and disease. **(A)** Human develop index (HDI) values for each administrative department are correlated with ethnic group population percentages for each department: Mestizo (orange), Afro-Colombian (blue), Indigenous (red). Each point represents a single department, and the linear trend is indicated for each ancestry along with 95% CI (shaded area). Pearson correlation coefficient and *p*-values are shown for each. **(B)** Correlations between prevalence and HDI are shown for six non-cancer conditions (top) and the eight cancers (bottom).

Health outcomes are strongly influenced by a variety of socioeconomic factors. The HDI is a composite index that is used to measure three key aspects of human development: life expectancy, education, and income. HDI in Colombia is related to demography with marked differences seen for the three ethnic groups studied here. The Mestizo population percentage is positively correlated with HDI, whereas the Afro-Colombian and Indigenous population percentages are negatively correlated with HDI (Figure 7A). Differences in the level of economic development and human capital captured by HDI may explain the differences in population health outcomes observed here. The prevalence of prioritized non-cancer conditions, Malaria in particular, tend to be negatively correlated with HDI, whereas cancer is uniformly positively correlated with HDI (Figure 7B). Clearly, human development and socioeconomic factors are strongly related with health outcomes in Colombia. Nevertheless, the patterns of genetic admixture observed for the Colombian population, which to some extent blur the genetic ancestry distinctions among ethnic groups, may obscure the relationship between socioeconomic factors, genetic ancestry, and disease risk in the country. This may explain the discordance seen between ethnicity and disease prevalence vs. ancestry and disease risk.

## Discussion

### Ethnicity vs. Genetic Ancestry in Colombia

Ethnic designations in Colombia are based on self-identification. In the 2005 census, individuals could choose from one of six ethnic categories, including Indigenous, *Rom*, three categories of Afro-Colombian groups, or none of the above (Supplementary Figure 1; Rojas Morales et al., 2007). For this study, we focused on the three largest ethnic groups: Mestizo, which corresponds to the “none of the above” option from the census, and may also include individuals who self-identify as white, Afro-Colombian, and Indigenous. The *Rom* category from the census corresponds to the recently recognized Roma population, whose presence in the Americas dates to Columbus’ earliest arrival but only make up 0.01% of the Colombian population. The small size of this ethnic group did not allow for sufficient resolution for the comparative analyses conducted here.

It is important to note that self-identified ethnicity is distinct from genetic ancestry as measured here. Colombians of all ethnicities show high levels of genetic admixture (Ruiz-Linares et al., 2014; Conley et al., 2017). Mestizos in particular have both substantial European and Native American genetic ancestry components and often show a small but not insubstantial African ancestry fraction as well. Even isolated Afro-Colombian and Indigenous Colombian communities show evidence of genetic admixture. Thus, while the ethnic categories used here are strongly correlated with genetic ancestry, they clearly do not capture the complexity of ancestry and admixture seen for the Colombian population. In addition to ancestry, self-identified ethnic categories also reflect individuals lived experiences, including many aspects of the environment and socioeconomic factors that can influence health outcomes. These distinctions between ethnicity and genetic ancestry may explain some of the differences observed between ethnic and genetic ancestry effects on disease prevalence and predicted risk observed here.

### Implications for Colombia: Precision Public Health

The strong relationship between ethnicity, ancestry, and health outcomes in Colombia support the adoption of the precision public health paradigm as a way to bring genomic medicine to the country. Precision public health is an alternative to precision medicine, as it entails focus on populations instead of individuals (Khoury et al., 2016, 2018; Weeramanthri et al., 2018). Precision public health relies on detailed health, demographic, and population genomic profiles of developing countries, which allows for the allocation of resources and efforts where they will be most effective. The hope is that an emphasis on population-level interventions will enable the emerging genomic technologies that underlie precision medicine to be efficiently implemented in developing countries. We recently showed how population genomic profiles can be used to guide pharmacogenetic decision making in Colombia in a way that targets resources to the populations, where they will generate the maximum benefit to health and the best return on investment (Nagar et al., 2019). The results reported here, particularly as they relate to the distinct health characteristics of different Colombian populations and regions, underscore the need to focus health intervention efforts in a population-specific way. To the extent that genomic approaches to healthcare are implemented in the country, they should be targeted to groups and areas where they can be expected to yield the most benefit and the least harm. This approach will need to be attuned to the differences between self-identified ethnicity and genetically measured ancestry demonstrated here.

### Limitations of the Study and Future Directions

One of the major limitations of this study is the lack of individual level health data for the cohort studied here. The ethnicity and disease prevalence data are reported at the level of administrative departments, which facilitates high-level comparisons but does not allow for detailed interrogation of the relationship between genetic ancestry and health. Future efforts to study the relationship between ethnicity, genetic ancestry, and health will be strengthened by the creation of population biobanks that include biological samples, deep phenotypic data, and electronic health records for individual participants. Biobanks also can and should include as many socioeconomic indicators as possible. Unfortunately, the goal of a country-level biobank, such as the United Kingdom Biobank or the United States All of Us project, remains out of reach for Colombia, at least for the time being. However, individual laboratories, including our own groups, are beginning to collaborate to marshal resources to create the kind of biobank data that will be needed to strengthen this line of research.

The use of different sources of data, both for disease prevalence values and for genome-wide genotyping, represents a potential source of bias in our study. However, this bias is likely mitigated by the choice of epidemiological databases and the methods for data harmonization used here. The disease prevalence databases were chosen as they are considered to be the most reliable epidemiological surveys of Colombia that are publicly available, with each database focused on a specific set of health conditions. For the two sources of genome-wide genotype data, we performed rigorous data quality control when harmonizing the data in an effort to avoid any potential bias. Visual inspection of the genomic relationships among individuals from each of the two genotype data sets does not point to any evidence of batch effects (Figures 2B,C).

Another potential limitation of this study relates to the predictive utility of PRS in the kind of diverse, admixed populations such as the Colombian cohort studied here. The vast majority of PRS have been developed and validated on cohorts with European genetic ancestry, and PRS may not transfer well across populations with distinct ancestry profiles. The PRS used here were mined from the NHGRI-EBI GWAS Catalog,^10^ and we only used PRS that were developed using cohorts that included two or more diverse ancestries in an effort to mitigate problems with cross-population portability. Nevertheless, the development and validation of PRS on admixed American populations, and for Colombian cohorts in particular, will certainly allow for more robust genetic risk prediction in the future.

Finally, it should be noted that the results here apply to a single admixed American population within a single Latin American country. Latin American populations are highly diverse and characterized by distinct combinations of African, European, and Native American ancestry (Bryc et al., 2010; Ruiz-Linares et al., 2014; Homburger et al., 2015; Norris et al., 2018, 2020). In addition, as we have shown here, Colombia itself is an ethnically diverse country with distinct ancestry profiles for different regions in the country (Rojas Morales et al., 2007; Rishishwar et al., 2015b; Conley et al., 2017; Nagar et al., 2019; Chande et al., 2020a). Thus, the connections between ancestry and health outcomes reported here may or may not apply to other countries or even to different populations sampled within Colombia. Future studies of this kind in other countries, or other populations within Colombia, can shed light on the extent to which the trends observed here are unique or shared among populations.

## Data Availability Statement

Colombian ethnicity, disease prevalence, and HDI data are made freely available online as described in the Materials and Methods section, and are provided here as Supplementary Material. WGG data reported in this study are available for research purposes by request to the corresponding authors. WGS data are made freely available without use restriction through the 1KGP.^11^

## Ethics Statement

The studies involving human participants were reviewed and approved by Ethics and Research Committee of the Universidad CES, Ethics Committee of the Universidad Tecnológica del Chocó, and Institutional Review Board of the Georgia Institute of Technology. The patients/participants provided their written informed consent to participate in this study.

## Author Contributions

AV-A, IJ, and JG conceived and designed the study. AC, SN, and LR performed all data analysis. LM-R, MM-R, AV-A, IJ, and JG supervised and managed all aspects of the project in Colombia and the United States. MM-R and JG acquired study subject samples. AC, SN, LR, and IJ prepared the figures and wrote the manuscript. All authors contributed to the article and approved the submitted version.

## Funding

AC, SN, LR, and IJ were supported by the IHRC-Georgia Tech Applied Bioinformatics Laboratory. LM-R was supported by the National Institutes of Health (NIH) Distinguished Scholars Program (DSP) and the Division of Intramural Research (DIR) of the National Institute on Minority Health and Health Disparities (NIMHD) at NIH (1ZIAMD000016 and 1ZIAMD000018). AV-A was supported by Fulbright Colombia. JG was supported by GenomaCES and Universidad CES.

## Conflict of Interest

The authors declare that the research was conducted in the absence of any commercial or financial relationships that could be construed as a potential conflict of interest.

## Publisher’s Note

All claims expressed in this article are solely those of the authors and do not necessarily represent those of their affiliated organizations, or those of the publisher, the editors and the reviewers. Any product that may be evaluated in this article, or claim that may be made by its manufacturer, is not guaranteed or endorsed by the publisher.

## Footnotes

^1^ https://www.genomaces.com/

^2^ https://www.chocogen.com/

^3^ https://cuentadealtocosto.org/

^4^ https://www.ins.gov.co/Direcciones/Vigilancia/Paginas/SIVIGILA.aspx

^5^ http://hdr.undp.org/en/content/human-development-index-hdi

^6^ http://hdr.undp.org/sites/default/files/hdr2020_technical_notes.pdf

^7^ https://globaldatalab.org/shdi/shdi/

^8^ https://www.illumina.com/products/by-type/microarray-kits/infinium-global-screening.html

^9^ https://www.illumina.com/products/by-type/microarray-kits/infinium-omni-express.html

^10^ https://www.ebi.ac.uk/gwas/

^11^ https://www.internationalgenome.org/

## References

[ref1] 1000 Genomes Project Consortium, Auton A. Brooks L. D. Durbin R. M. Garrison E. P. Kang H. M.. (2015). A global reference for human genetic variation. Nature 526, 68–74. doi: 10.1038/nature15393, PMID: 26432245 PMC4750478

[ref2] Alexander D. H. Novembre J. Lange K. (2009). Fast model-based estimation of ancestry in unrelated individuals. Genome Res. 19, 1655–1664. doi: 10.1101/gr.094052.109, PMID: 19648217 PMC2752134

[ref3] Bigdeli T. B. Genovese G. Georgakopoulos P. Meyers J. L. Peterson R. E. Iyegbe C. O.. (2020). Contributions of common genetic variants to risk of schizophrenia among individuals of African and Latino ancestry. Mol. Psychiatry 25, 2455–2467. doi: 10.1038/s41380-019-0517-y, PMID: 31591465 PMC7515843

[ref4] Bryc K. Velez C. Karafet T. Moreno-Estrada A. Reynolds A. Auton A.. (2010). Colloquium paper: genome-wide patterns of population structure and admixture among Hispanic/Latino populations. Proc. Natl. Acad. Sci. U. S. A. 107(Suppl. 2), 8954–8961. doi: 10.1073/pnas.0914618107, PMID: 20445096 PMC3024022

[ref26] Bustamante C. D. Burchard E. G. De la Vega F. M. (2011). Genomics for the world. Nature 475, 163–165. doi: 10.1038/475163a 21753830 PMC3708540

[ref5] Bustamente J. Jaramillo C. Cárdenas M. A. Acevedo L. Freire E. Gutiérrez C. (2012). Atlas Estadistico. Bogotá: Departamento Administrativo Nacional de Estadistica.

[ref6] Chande A. T. Rishishwar L. Ban D. Nagar S. D. Conley A. B. Rowell J.. (2020a). The phenotypic consequences of genetic divergence between admixed latin american populations: Antioquia and Choco, Colombia. Genome Biol. Evol. 12, 1516–1527. doi: 10.1093/gbe/evaa154, PMID: 32681795 PMC7513793

[ref27] Chande A. T. Rishishwar L. Conley A. B. Valderrama-Aguirre A. Medina-Rivas M. A. Jordan I. K. (2020b). Ancestry effects on type 2 diabetes genetic risk inference in Hispanic/Latino populations. BMC Med. Genet. 21(Suppl 2):132. doi: 10.1186/s12881-020-01068-0 32580712 PMC7315475

[ref28] Chande A. T. Rowell J. Rishishwar L. Conley A. B. Norris E. T. Valderrama-Aguirre A.. (2017). Influence of genetic ancestry and socioeconomic status on type 2 diabetes in the diverse Colombian populations of Choco and Antioquia. Sci. Rep. 7:17127. doi: 10.1038/s41598-017-17380-4 29215035 PMC5719455

[ref7] Chande A. T. Wang L. Rishishwar L. Conley A. B. Norris E. T. Valderrama-Aguirre A.. (2018). GlobAl distribution of genetic traits (GADGET) web server: polygenic trait scores worldwide. Nucleic Acids Res. 46, W121–W126. doi: 10.1093/nar/gky415, PMID: 29788182 PMC6031022

[ref8] Chang C. C. Chow C. C. Tellier L. C. Vattikuti S. Purcell S. M. Lee J. J. (2015). Second-generation PLINK: rising to the challenge of larger and richer datasets. GigaScience 4:7. doi: 10.1186/s13742-015-0047-8, PMID: 25722852 PMC4342193

[ref29] Collins F. S. Varmus H. (2015). A new initiative on precision medicine. N. Engl. J. Med. 372, 793–795. doi: 10.1056/NEJMp1500523 25635347 PMC5101938

[ref9] Conley A. B. Rishishwar L. Norris E. T. Valderrama-Aguirre A. Marino-Ramirez L. Medina-Rivas M. A.. (2017). A comparative analysis of genetic ancestry and admixture in the Colombian populations of Choco and Medellin. G3 7, 3435–3447. doi: 10.1534/g3.117.1118, PMID: 28855283 PMC5633392

[ref10] Homburger J. R. Moreno-Estrada A. Gignoux C. R. Nelson D. Sanchez E. Ortiz-Tello P.. (2015). Genomic insights into the ancestry and demographic history of South America. PLoS Genet. 11:e1005602. doi: 10.1371/journal.pgen.1005602, PMID: 26636962 PMC4670080

[ref11] Howie B. Fuchsberger C. Stephens M. Marchini J. Abecasis G. R. (2012). Fast and accurate genotype imputation in genome-wide association studies through pre-phasing. Nat. Genet. 44, 955–959. doi: 10.1038/ng.2354, PMID: 22820512 PMC3696580

[ref12] Howie B. Marchini J. Stephens M. (2011). Genotype imputation with thousands of genomes. G3 1, 457–470. doi: 10.1534/g3.111.001198, PMID: 22384356 PMC3276165

[ref30] Jameson J. L. Longo D. L. (2015). Precision medicine--personalized, problematic, and promising. N. Engl. J. Med. 372, 2229–2234. doi: 10.1056/NEJMsb1503104 26014593

[ref31] Jordan I. K. (2016). The Columbian exchange as a source of adaptive introgression in human populations. Biol. Direct 11:17. doi: 10.1186/s13062-016-0121-x 27038633 PMC4818900

[ref13] Khoury M. J. Bowen M. S. Clyne M. Dotson W. D. Gwinn M. L. Green R. F.. (2018). From public health genomics to precision public health: a 20-year journey. Genet. Med. 20, 574–582. doi: 10.1038/gim.2017.211, PMID: 29240076 PMC6384815

[ref14] Khoury M. J. Iademarco M. F. Riley W. T. (2016). Precision public health for the era of precision medicine. Am. J. Prev. Med. 50, 398–401. doi: 10.1016/j.amepre.2015.08.031, PMID: 26547538 PMC4915347

[ref32] Martin A. R. Gignoux C. R. Walters R. K. Wojcik G. L. Neale B. M. Gravel S.. (2017). Human demographic history impacts genetic risk prediction across diverse populations. Am. J. Hum. Genet. 100, 635–649. doi: 10.1016/j.ajhg.2017.03.004 28366442 PMC5384097

[ref33] Martin A. R. Kanai M. Kamatani Y. Okada Y. Neale B. M. Daly M. J. (2019). Clinical use of current polygenic risk scores may exacerbate health disparities. Nat. Genet. 51, 584–591. doi: 10.1038/s41588-019-0379-x 30926966 PMC6563838

[ref15] Medina-Rivas M. A. Norris E. T. Rishishwar L. Conley A. B. Medrano-Trochez C. Valderrama-Aguirre A.. (2016). Choco, Colombia: a hotspot of human biodiversity. Rev. Biodivers. Neotrop. 6, 45–54. doi: 10.18636/bioneotropical.v6i1.341, PMID: 27668076 PMC5033504

[ref16] Nagar S. D. Moreno A. M. Norris E. T. Rishishwar L. Conley A. B. O'Neal K. L.. (2019). Population pharmacogenomics for precision public health in Colombia. Front. Genet. 10:241. doi: 10.3389/fgene.2019.00241, PMID: 30967898 PMC6439339

[ref17] Norris E. T. Rishishwar L. Chande A. T. Conley A. B. Ye K. Valderrama-Aguirre A.. (2020). Admixture-enabled selection for rapid adaptive evolution in the Americas. Genome Biol. 21:29. doi: 10.1186/s13059-020-1946-2, PMID: 32028992 PMC7006128

[ref18] Norris E. T. Wang L. Conley A. B. Rishishwar L. Marino-Ramirez L. Valderrama-Aguirre A.. (2018). Genetic ancestry, admixture and health determinants in Latin America. BMC Genomics 19(Suppl. 8):861. doi: 10.1186/s12864-018-5195-7, PMID: 30537949 PMC6288849

[ref19] Pato M. T. Sobell J. L. Medeiros H. Abbott C. Sklar B. M. Buckley P. F.. (2013). The genomic psychiatry cohort: partners in discovery. Am. J. Med. Genet. B Neuropsychiatr. Genet. 162B, 306–312. doi: 10.1002/ajmg.b.32160, PMID: 23650244 PMC3729260

[ref34] Petrovski S. Goldstein D. B. (2016). Unequal representation of genetic variation across ancestry groups creates healthcare inequality in the application of precision medicine. Genome Biol. 17:157. doi: 10.1186/s13059-016-1016-y 27418169 PMC4944427

[ref35] Popejoy A. B. Fullerton S. M. (2016). Genomics is failing on diversity. Nature 538, 161–164. doi: 10.1038/538161a 27734877 PMC5089703

[ref20] R Core Team (2020). R: A Language and Environment for Statistical Computing. Vienna, Austria: R Foundation for Statistical Computing.

[ref36] Rishishwar L. Conley A. B. Vidakovic B. Jordan I. K. (2015a). A combined evidence Bayesian method for human ancestry inference applied to Afro-Colombians. Gene 574, 345–351. doi: 10.1016/j.gene.2015.08.015 26275940

[ref21] Rishishwar L. Conley A. B. Wigington C. H. Wang L. Valderrama-Aguirre A. Jordan I. K. (2015b). Ancestry, admixture and fitness in Colombian genomes. Sci. Rep. 5:12376. doi: 10.1038/srep12376, PMID: 26197429 PMC4508918

[ref22] Rojas Morales E. Fernández Ayala P. J. Gallo Mejía H. Serna Rios C. Poveda Gómez M. (2007). Colombia Una Nación Multicultural: Su Diversidad Étnia. Bogotá: DANE.

[ref23] Ruiz-Linares A. Adhikari K. Acuna-Alonzo V. Quinto-Sanchez M. Jaramillo C. Arias W.. (2014). Admixture in Latin America: geographic structure, phenotypic diversity and self-perception of ancestry based on 7,342 individuals. PLoS Genet. 10:e1004572. doi: 10.1371/journal.pgen.1004572, PMID: 25254375 PMC4177621

[ref24] Weeramanthri T. S. Dawkins H. J. S. Baynam G. Bellgard M. Gudes O. Semmens J. B. (2018). Editorial: precision public health. Front. Public Health 6:121. doi: 10.3389/fpubh.2018.00121, PMID: 29761096 PMC5937027

[ref25] Wickham H. (2016). Ggplot2: Elegant Graphics for Data Analysis. New York: Springer-Verlag.

