# Supplementary material for: The Impact of Ethnicity and Genetic Ancestry on Disease Prevalence and Risk in Colombia
Source: Front Genet. 2021 Sep 17;12:690366. doi: 10.3389/fgene.2021.690366 (PMC8507149; doi:10.3389/fgene.2021.690366)
Supplement: Supplementary file 1 [file Data_Sheet_1.PDF]

Supplementary material for

The impact of ethnicity and genetic ancestry on disease prevalence and risk in Colombia

Aroon T. Chande, Shashwat D. Nagar, Lavanya Rishishwar, Leonardo Mariño-Ramírez, Miguel A. Medina-Rivas, Augusto E. Valderrama-Aguirre, I. King Jordan, Juan Esteban Gallo

Contents

1 Supplementary Figures 2

Supplementary Figure 1. 2

Supplementary Figure 2. 3

1 Supplementary Figures

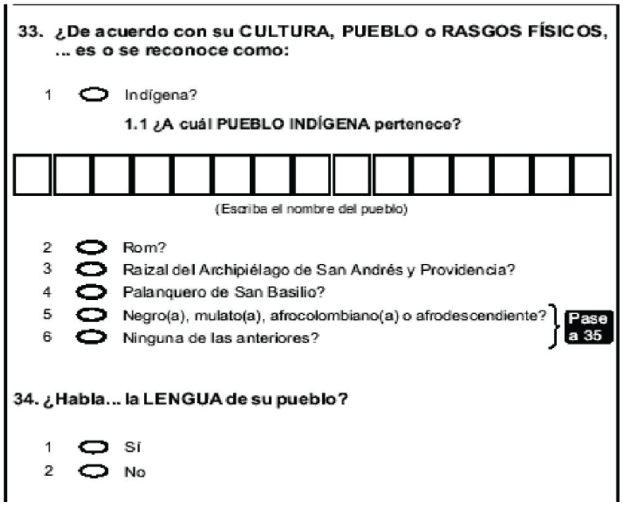
33. ¿De acuerdo con su CULTURA, PUEBLO o RASGOS FÍSICOS, ... es o se reconoce como:

1 Indígena?

1.1 ¿A cuál PUEBLO INDÍGENA pertenece?

(Escriba el nombre del pueblo)

2 Rom?
3 Raizal del Archipiélago de San Andrés y Providencia?
4 Palanquero de San Basilio?
5 Negro(a), mulato(a), afrocolombiano(a) o afrodescendiente?
6 Ninguna de las anteriores?

Pase a 35

34. ¿Habla... la LENGUA de su pueblo?

1 Sí
2 No

Supplementary Figure 1. Colombian census questionnaire used by DANE showing the ethnic group identities. Question #33 asks (translation) "According to your culture, people or physical traits, you are or are recognized as:" Option #1 corresponds to Indigenous ethnicity, and the follow up question #1.1 asks about specific tribal or community affiliation. Options #3, #4, and #5 all correspond to distinct Afro-Colombian groups or identities. The final option #6 is translated as "None of the above" and corresponds to the majority Mestizo ethnic group.

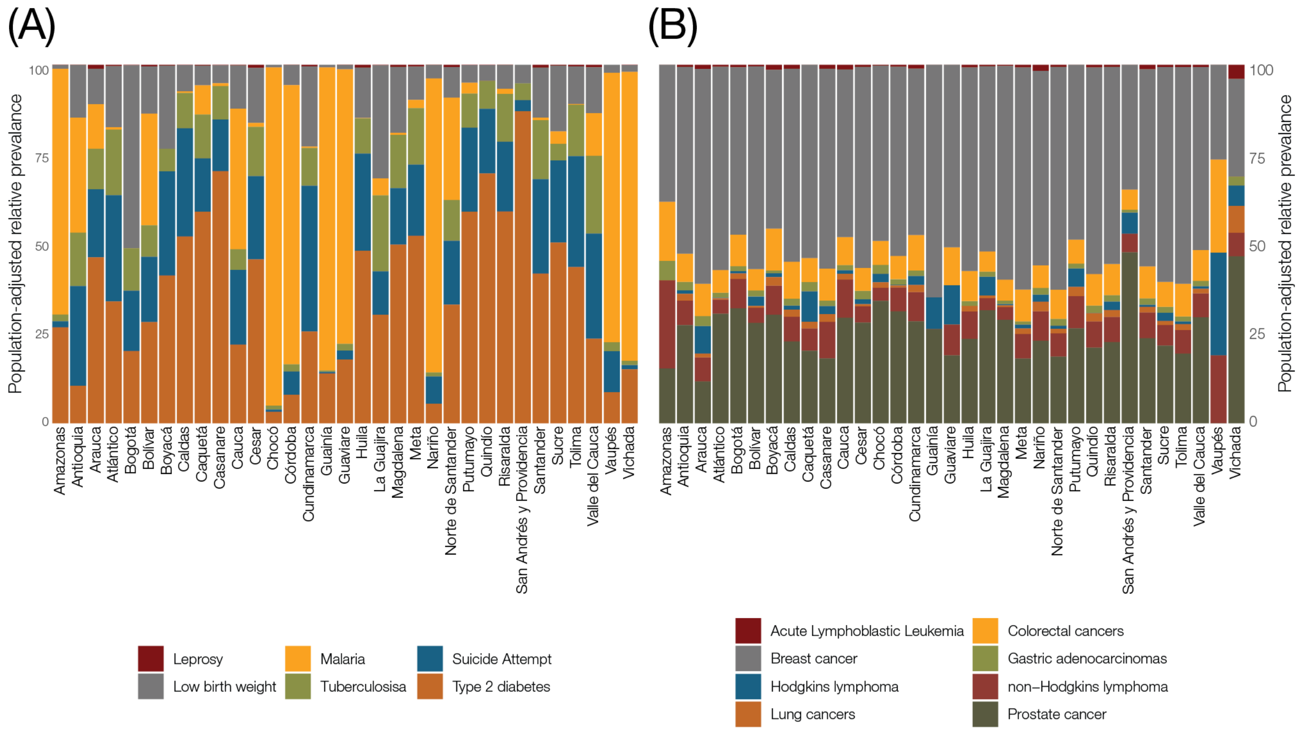

Supplementary Figure 2. Population-adjusted relative prevalence values are shown across all Colombian administrative departments for (A) the six prioritized non-cancer conditions and (B) the eight common cancers. Observed disease prevalence values are normalized to 100 for all departments to yield relative disease prevalence values for each department.
